# Comparison of the different voxel sizes in the estimation of peri-implant fenestration defects using cone beam computed tomography: an ex vivo study

**DOI:** 10.1186/s40729-020-00254-2

**Published:** 2020-10-02

**Authors:** Mehmet Hakan Kurt, Nilsun Bağış, Cengiz Evli, Cemal Atakan, Kaan Orhan

**Affiliations:** 1 Department of Dentomaxillofacial Radiology, Faculty of Dentistry, Ankara University, Ankara, Turkey; 2 Dentistry Department of Periodontology, Ankara University, Ankara, Turkey; 3 Faculty of Science Department of Statistics, Ankara University, Ankara, Turkey; 4 Medical Design Application and Research Center (MEDITAM), Ankara University, Ankara, Turkey

**Keywords:** Cone beam computed tomography, Peri-implant fenestrations, Implant, Voxel size

## Abstract

**Background:**

To examine the influence of voxel sizes to detect of peri-implant fenestration defects on cone beam computed tomography (CBCT) images.

**Materials and methods:**

This study performed with three sheep heads both maxilla and mandible and two types of dental implant type 1 zirconium implant (Zr^40^) (*n* = 6) and type 2 titanium implant (Ti^22^) (*n* = 10). A total of 14 peri-implant fenestrations (8 buccal surfaces, 6 palatal/lingual surface) were created while 18 surfaces (8 buccal, 10 palatal/lingual) were free of fenestrations. Three observers have evaluated the images of fenestration at each site. Images obtained with 0.75 mm^3^, 0.100 mm^3^, 0.150 mm^3^, 0.200 mm^3^, and 0.400 mm^3^ voxel sizes. For intra- and inter-observer agreements for each voxel size, Kappa coefficients were calculated.

**Results:**

Intra- and inter-observer kappa values were the highest for 0.150 mm^3^, and the lowest in 0.75 mm^3^ and 0.400 mm^3^ voxel sizes for all types of implants. The highest area under the curve (AUC) values were found higher for the scan mode of 0.150 mm^3^, whereas lower AUC values were found for the voxel size for 0.400 mm^3^. Titanium implants had higher AUC values than zirconium with the statistical significance for all voxel sizes (*p* ≤ 0.05).

**Conclusion:**

A voxel size of 0.150 mm^3^ can be used to detect peri-implant fenestration bone defects. CBCT is the most reliable diagnostic tool for peri-implant fenestration bone defects.

## Introduction

Oral implants have become the most popular treatment choice for the replacement of missing teeth since introduced by Brånemark [1]. The “gold standard” material is titanium and its biomedical alloys, due to its long-term clinical survival rates for endosseous dental implants [2–4]. Even though titanium is the gold standard for dental implants various materials involving gold, stainless steel, and cobalt-chromium have been used in the dental implant industry. These materials are withdrawn from the oral implant industry because of their adverse side effects and had a low long-term survival rate [5, 6]. Zirconium is an alternative material because of its tooth-like color and its osseointegration potential. Besides, less plaque accumulation on zirconium than on titanium surfaces makes this material more attractive for the implant industry [7–9].

The long-term success of dental implants established on the health of soft and hard tissues [10]. Moreover, the most important issue is to have a sufficient amount of cortical bone around the implant because of the primary stability and osseointegration for the success of the implant treatment [11]. Additionally, inadequate cortical plate or amount of the bone revealed the risk of bone defects such as fenestration and dehiscence around the implants. Because of this situation, the success of implant treatment increases dramatically [12]. Bone defects that occur around dental implants may adversely affect gum health and cause some aesthetic problems. More importantly, implant failures may occur in the long-term. Thus, early detection of bone defects around dental implants is very important to prevent the problems [13]. Intraoral imaging modality can demonstrate the presence of bone in the mesial and distal region and also in the implant-bone inter-face. Bone loss after the initial insertion is most commonly seen in the buccal region because the bone is thinner in this area surrounding the implant [14]. Intraoral radiography techniques are two-dimensional imaging methods and have some limitations such as superimposition or distortion of anatomical structures. The visualization of buccolingual walls cannot demonstrate these methods [14, 15].

CBCT has been suggested as an alternative tool in implant dentistry for many procedures, including linear measurements of alveolar bone, graft planning, following-up after implant placement, or three dimensional (3D) evaluation of bone defects [16, 17]. It eliminates all disadvantages of two dimensional (2D) images such as superimposition, image distortion, or imaging buccolingual aspects of the bone [12, 13, 18, 19]. CBCT images allow the examination of images in all planes with submillimetric resolution and without distortion. This allows a more accurate examination of the bone defects around the implants [16, 20–22]. On the other hand, metallic objects used in dentistry such as amalgam or titanium implants can induce two kinds of artifacts described as beam hardening and streaking in the CBCT images. Both of these artifacts affect the visualization of areas and decrease the image quality dramatically.

Dark areas adjacent to high-density structures are called Beam hardening artifacts. This situation is explained with high-density materials that absorb the low-energy X-ray photons [23, 24]. Streak artifacts occur due to scattering radiation from metallic objects, and they are linked to the high-density objects and are seen as linear hyper densities extending along the width of the field [20, 25]. Zirconium implants create more artifacts than titanium implants in CBCT images [26]. Moreover, inaccuracies of inter-proximal peri-implant defect detection on CBCT are more definite in zirconium implants compared with titanium implants [27]. Hence, the results obtained from the studies with titanium implants cannot be applied to zirconium implants [28].

A voxel is the smallest part of a 3D image and it is isotropic in CBCT images. The selection of voxel size can be useful for the detection of peri-implant bone defects. This improves spatial resolution. In other words, increment of the spatial resolution can improve the ability of examination very small distances of the objects [20, 29]. However, there is no specific protocol described for the CBCT examination of peri-implant bone defects with different voxel size variation [30].

Hence, this study aimed to examine the effect of voxel sizes to detect of peri-implant fenestration defects on CBCT images.

## Material and methods

**Fig. 1:**
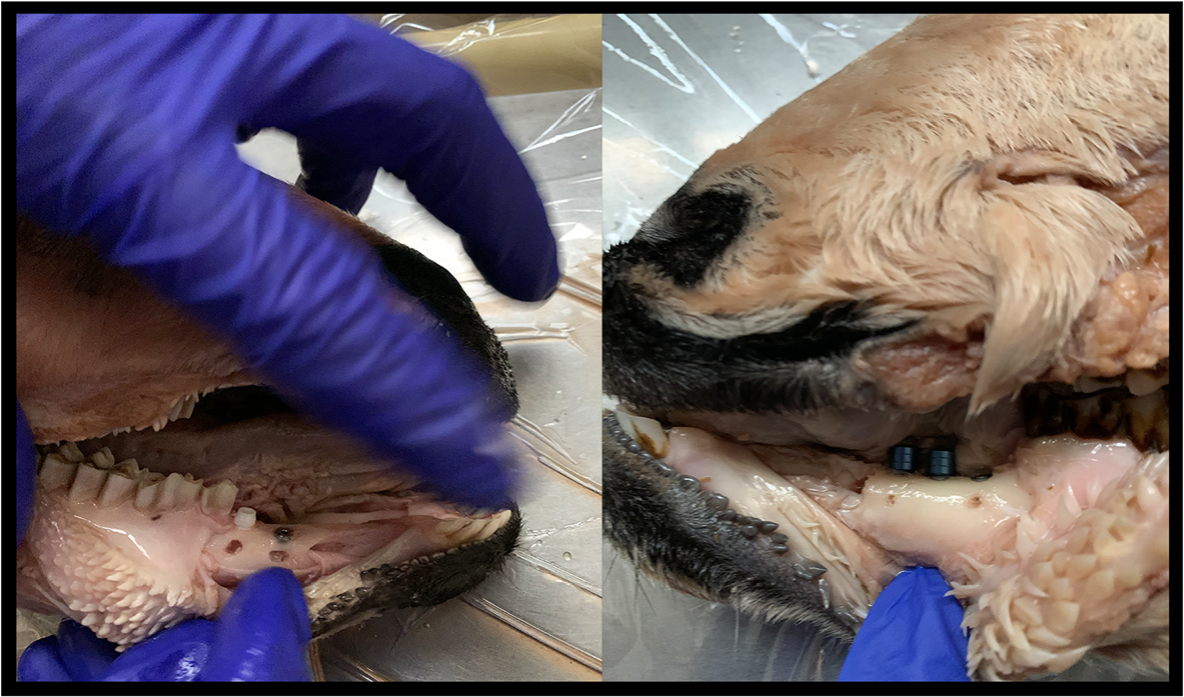
Photo of the positioned implants and fenestration defects in the sheep’s jaw

This study performed with three sheep heads both maxilla and mandible and two types of dental implant (type 1 zirconium implant (*n* = 6) (Zeramex XT, Miami, FL, USA), type 2 titanium implant (*n* = 10) (DAND Dental implants, D.A.N.D. Metal Industries Ltd., Yavne, Israel). One periodontology specialist with experience placed all dental implants into the sheep heads (Fig. 1). After placed dental implants, the same operator creates artificial defects around dental implants. These simulating defects were created with high-speed equipment copious air/water spray to the cervical portion of the implant. Round shaped diamond burs (KG Sorensen, Zenith Dental Aps, Agerskov, Denmark) with a 3-mm diameter were used in buccal or palatal/lingual surfaces. In total 14 fenestrations (8 buccal surfaces, 6 palatal/lingual surface) were created. Eighteen surfaces (8 buccal, 10 palatal/lingual) were free of fenestrations. All fenestrations except one titanium and one zirconium implants created separately buccal or palatal/lingual surfaces; this fenestration in these implants created buccal and lingual/palatal surfaces. These bone defects were created without knowing the depth. After creating the defects around dental implants, the mucoperiosteal flap was repositioned carefully. These fenestration defects noted by the same operator to be used as a gold standard for the image evaluations.

### Radiographic imaging

**Fig. 2:**
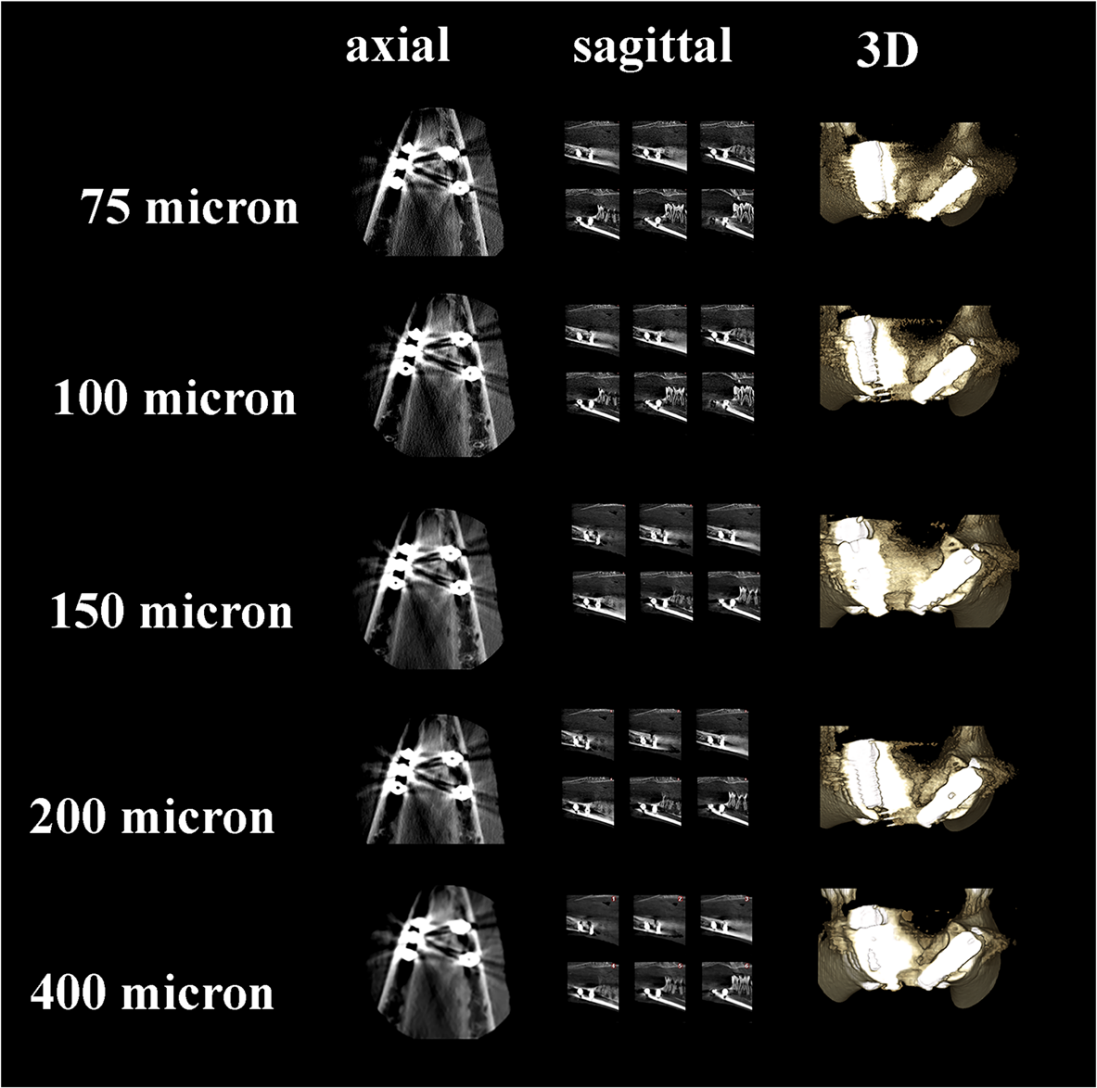
Axial, sagittal, and 3D slices from CBCT images of titanium implants with simulated peri-implant fenestration defects with five voxel sizes

**Fig. 3:**
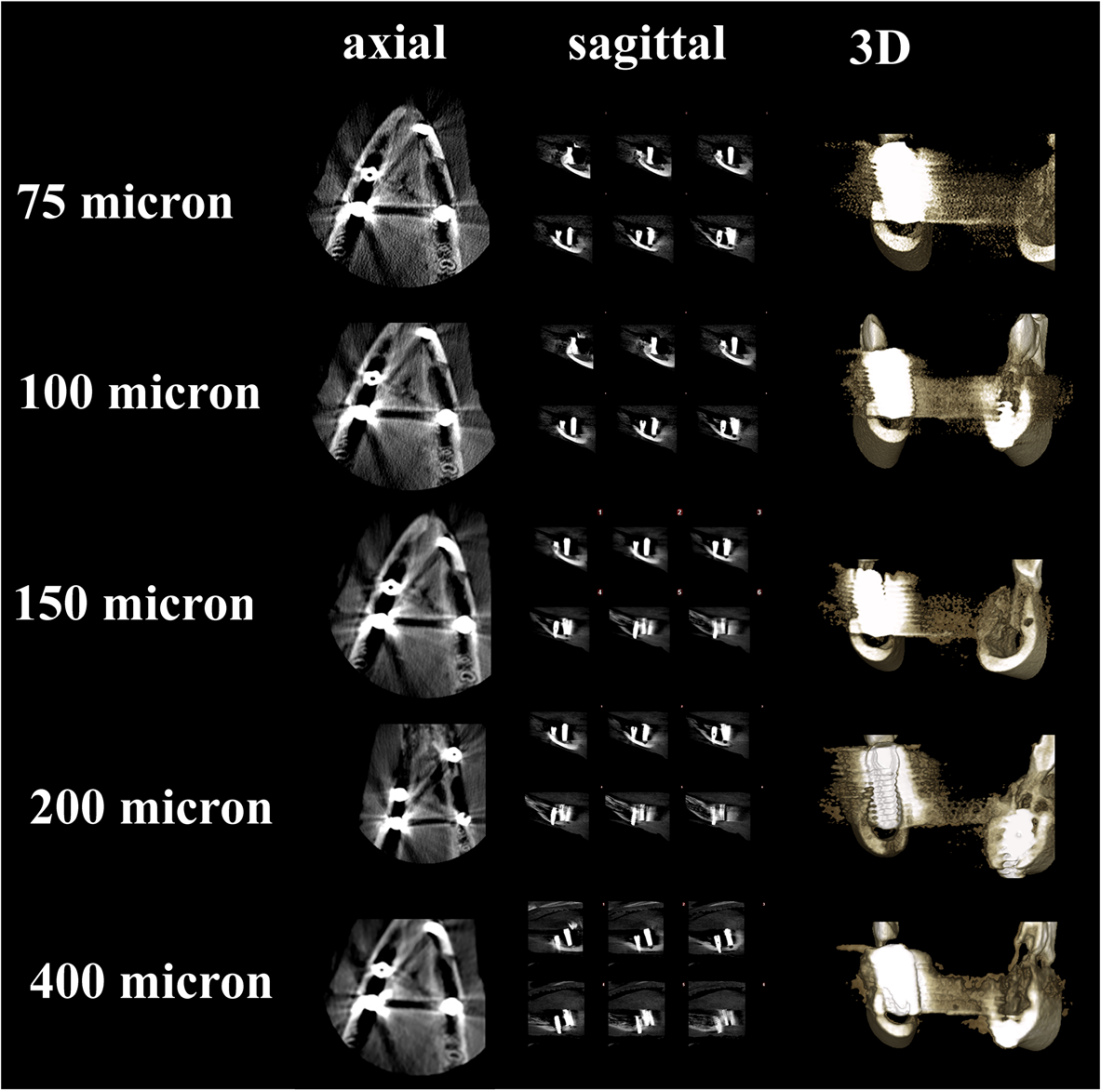
Axial, sagittal and 3D slices from CBCT images of zirconium implants with simulated peri-implant fenestration defects with five voxel sizes

Sheep heads were fixed to the machine to provide standardization during CBCT scanning. All images obtained by Planmeca Promax 3D Max CBCT unit (Planmeca Oy, Helsinki, Finland) with following voxel size and parameters 0.075 mm^3^ (90 kV, 10 mA, 15 sn, 5 × 5.5 FOV, DAP value 730 mGy × cm^2^), 0.100 mm^3^ (90 kV, 10 mA, 12 sn, 5 × 5.5 FOV, DAP value 632 mGy × cm^2^), 0.150 mm^3^ (90 kV, 10 mA, 12 sn, 5 × 5.5 FOV, DAP value 584 mGy × cm^2^), 0.200 mm^3^ (90 kV, 10 mA, 12 sn, 5 × 5.5 FOV, DAP value 584 mGy × cm^2^), and 0.400 mm^3^ (90 kV, 10 mA, 6 sn, 5 × 5.5 FOV, DAP value 293 mGy × cm^2^). The images of fenestrations around titanium and zirconium implants obtained with five voxel scan modes are demonstrated in Figs. 2 and 3.

After image acquisition, all measurements performed on a 21.3-in. flat panel color active matrix thin film transistor (TFT) medical display (NEC MultiSync MD215MG, München, Germany) with a resolution of 2048 × 2560 at 75 Hz and 0.17-mm dot pitch operated at 11.9 bits. Three observers evaluated all CBCT scans.

### Image analysis

Each scan was evaluated by 3 observers with different times of experience (ranged from 1.8 years to 4.7 years) on CBCT imaging. The observers were blinded to the clinical situation regarding the defect size and locations. All evaluations were carried out with the Planmeca CBCT unit’s own software (Romexis 4.3 Planmeca Oy, Helsinki, Finland). The observers were permitted to use both image enhancement and processing functions of the software. For CBCT, the images were anonymized while evaluating the fenestrations. All observers were trained to use the software and calibrate for the appearance of fenestrations.

For all imaging methods, a five-point scale was used to assess each fenestration visibility as (1) definitely absent; (2) probably absent; (3) unsure; (4) probably present; (5) definitely present. The observers were asked to define the fenestrations in CBCT images. Each image set was evaluated by 1 week intervals, and all evaluations were repeated 2 months after the initial examinations.

### Examiner reliability and statistical analysis

Hence, in this study, AUC-ROC and Kappa coefficients were calculated to determine intra- and inter-observer agreements by different voxel sizes. Cohen’s Kappa values were clarified as κ < 0.00, no agreement; κ = 0.00–0.20, poor agreement; *κ* = 0.21–0.40, fair agreement; κ = 0.41–0.60, moderate agreement; κ = 0.61–0.80, good agreement; and κ = 0.81–1.00, very good agreement [31].

AUC values were clarified as AUC = 0.5: no discrimination; 0.5 < AUC < 0.7: poor discrimination; 0.7 ≤ AUC < 0.8: acceptable discrimination; 0.8 ≤ AUC < 0.9: excellent discrimination; AUC ≥ 0.9: outstanding discrimination [32].

A receiver operating characteristic curve, or ROC curve, is a graphical plot that illustrates the diagnostic ability of a binary classifier system as its discrimination threshold is varied. The ROC curve is created by plotting the true positive rate (TPR) against the false-positive rate (FPR) at various threshold settings. The true-positive rate is also known as sensitivity. The false-positive rate is also known as (1—specificity). AUC-ROC curve is a performance measurement for classification problem at various thresholds settings. ROC is a probability curve and AUC represents degree or measure of separability. It tells how much model is capable of distinguishing between classes. The higher the AUC, the better the model is at predicting 0 s as 0 s and 1 s as 1 s. By analogy, the higher the AUC, the better the model is at distinguishing between patients with disease and no disease. Statistical analyses were done for each image type, observer, and reading using the Mann-Whitney *U* test and the Kruskal-Wallis H test to determine the differences between the groups. Differences of a *p* value of less than 0.05 considered statistically significant.

## Results

Intra-observer and inter-observer kappa coefficients are shown in Tables 1 and 2, respectively.

**Table 1:** Intraobserver kappa values calculated by scan modes and implant types for each observer

	Kappa values (Se)					
Titanium (voxel size)	Obs 1 (1st reading-2nd reading)	*p* values	Obs 2 (1st reading-2nd reading)	p- values	Obs 3 (1st reading-2nd reading)	p-values
0.075 mm^3^	0.667 (0.073)	*p* > 0.005	0.771 (0.085)	*p* > 0.005	0.552(0.170)	*p* > 0.005
0.100 mm^3^	0.778 (0.158)	*p* > 0.005	0.789 (0.089)	*p* > 0.005	0.625(0.171)	*p* > 0.005
0.150 mm^3^	0.830(0.114)	*p* > 0.005	1.00(0.000)	*p* > 0.005	0.842(0.101)	*p* > 0.005
0.200 mm^3^	0.830(0.114)	*p* > 0.005	1.00(0.000)	*p* > 0.005	0.816(0.184)	*p* > 0.005
0.400 mm^3^	0.671(0.108)	*p* > 0.005	0.689(0.089)	*p* > 0.005	0.625(0.170)	*p* > 0.005
	Kappa values (Se)					
Zirconium (voxel size)	Obs 1 (1st reading-2nd reading)	*p* values	Obs 2 (1st reading-2nd reading)	*p* values	Obs 3 (1st reading-2nd reading)	*p* values
0.075 mm^3^	0.600(0.343)	*p* > 0.005	0.600(0.343)	*p* > 0.005	0.600(0.343)	*p* > 0.005
0.100 mm^3^	0.600(0.343)	*p* > 0.005	0.600(0.343)	*p* > 0.005	0.600(0.343)	*p* > 0.005
0.150 mm^3^	0.771(0.143)	*p* > 0.005	0.750(0.084)	*p* > 0.005	0.671(0.108)	*p* > 0.005
0.200 mm^3^	0.600(0.343)	*p* > 0.005	0.600(0.343)	*p* > 0.005	0.600(0.343)	*p* > 0.005
0.400 mm^3^	0.385(0.297)	*p* > 0.005	− 0.143(0.100)	*p* > 0.005	0.600(0.343)	*p* > 0.005

*Abbreviations*: *SE* standart error, *1st* first, *2nd* second readings

**Table 2:** Inter-observer kappa coefficients value and standard error according to scan modes and implant types for 1st and 2nd readings

		obs. 1–2			obs 1–3			obs 2–3		
Voxel size	Implant Type	1st reading κ - SE	2nd reading κ - SE	*p* values (≤ 0.005)	1st reading κ - SE	2nd reading κ - SE	*p* values (≤ 0.005)	1st reading κ - SE	2nd reading κ - SE	*p* values (≤ 0.005)
1 (a)	1	0.644(0.162)	0.727(0.146)	a-c	0.647(0.158)	0.690(0.083)	a-c	0.647(0.158)	0.727(0.146)	a-c
	2	0.600(0.343)	0.600(0.343)	a-d-e	0.600(0.343)	0.600(0.343)	a-d-e	0.600(0.343)	0.600(0.343)	a-d-e
2 (b)	1	0.743(0.134)	0.813(0.184)	b-c	0.739(0.140)	0.739(0.140)	b-c	0.690(0.083)	0.739(0.140)	b-c
	2	0.600(0.343)	0.600(0.343)	b-d-e	0.613(0.184)	0.600(0.343)	b-d-e	0.600(0.343)	0.600(0.343)	b-d-e
3 (c)	1	1.000(0.000)	1.000(0.000)	a-b-d-e	0.743(0.134)	0.846(0.131)	a-b-d-e	0.909(0.089)	0.812(0.127)	a-b-d-e
	2	0.712(0.078)	0.667(0.128)	c-d-e	0.689(0.084)	0.613(0.184)	c-d-e	0.634(0.054)	0.671(0.058)	c-d-e
4 (d)	1	0.846(0.131)	0.812(0.131)	d-c	0.777(0.184)	0.813(0.184)	d-c	0.812(0.131)	0.709(0.131)	d-c
	2	0.624(0.043)	0.667(0.128)	a-b-c-d	0.671(0.058)	0.700(0.056)	a-b-c-d	0.600(0.343)	0.739(0.140)	a-b-c-d
5 (e)	1	0.612(0.067)	0.668(0.124)	e-c	0.671(0.058)	0.667(0.128)	e-c	0.678(0.089)	0.600(0.124)	e-c
	2	0.385(0.297)	0.600(0.343)	a-b-c-e	0.385(0.297)	0.600(0.343)	a-b-c-e	0.385(0.297)	0.600(0.343)	a-b-c-e

*Abbreviations*: *SE* standart error, *1st* first, *2nd* second readings; voxel sizes: (1) 0.075 mm^3^, (2) 0.100 mm^3^, (3) 0.150 mm^3^, (4) 0.200 mm^3^, (5) 0.400 mm^3^; implant types: (1) titanium implant, (2) zirconium implant

Same letters indicate statistical significance **≤** 0.005

The Kappa values of intra-observer agreements for the titanium implants according to voxel sizes were the highest for 0.150 mm^3^, 0.200 mm^3^ voxel sizes for all observers (very good agreement) while for the zirconium implants the kappa values were varied according to voxel sizes with the highest kappa value for 0.150 mm^3^ (good agreement) (Table 1). There are no significant differences between first and second readings for all observers (> 0.05).

Table 2 shows inter-observer kappa values for first and second readings of all observers; for zirconium implants, the lowest values achieved with 0.075 and 0.400 mm^3^ voxel sizes (poor to moderate). For titanium implants, the highest kappa value achieved for 0.150 mm^3^ voxel size (good to very good agreement) with a statistically significant difference for all inter-observer evaluations (Table 2). For the zirconium implant, there was also a statistically significant difference for 0.200 and 0.400 mm^3^ for all inter-observer evaluations (*p* ≤ 0.05).

AUC values were evaluated for each voxel size, implant type, and observer by using two readings (Table 3). While AUC values for titanium implants from ranged from 0.771 to 0.826 in 0.075 mm^3^ voxel sizes (acceptable to excellent discrimination), for 0.100 mm^3^ 0.771 to 0.875 (acceptable to excellent discrimination), for 0.150 mm^3^ ranged from 0.785 to 0.917 (excellent to outstanding discrimination). AUC values 0.200 mm^3^ ranged from 0.771 to 0.875 and for 0.400 mm^3^ ranged from 0.726 to 0.819 (acceptable to excellent discrimination). In summary, titanium implants were found to have higher AUC values than zirconium implants with a significant difference for all voxel sizes and observers (*p* ≤ 0.05).

**Table 3:** AUC values according to scan modes and implant types for 1st and 2nd readings of the observers

		Observer 1			Observer 2			Observer 3		
Voxel size	Implant type	1st reading AUC–SE	2nd reading AUC–SE	*p* values (≤ 0.005)	1st reading AUC–SE	2nd reading AUC–SE	*p* values (≤ 0.005)	1st reading AUC–SE	2nd reading AUC–SE	*p* values (≤ 0.005)
1	1 (a)	0.771–0.102	0.826–0.090	a-b	0.819–0.090	0.826–0.091	a-b	0.826–0.091	0.826–0.089	a-b
	2 (b)	0.580–0.106	0.583–0.206		0.208–0.215	0.333–0.217		0.500–0.219	0.625–0.240	
2	1 (a)	0.816–0.093	0.826–0.090	a–b	0.799–0.093	0.812–0.093	a-b	0.875–0.080	0.771–0.101	a-b
	2 (b)	0.667–0.122	0.500–0.219		0.417–0.309	0.417–0.230		0.458–0.233	0.625–0.240	
3	1 (a)	0.819–0.093	0.917–0.066	a-b	0.823–0.092	0.785–0.101	a-b	0.833–0.090	0.875–0.139	a-b
	2 (b)	0.691–0.097	0.500–0.219		0.417–0.309	0.208–0.215		0.500–0.219	0.667–0.248	
4	1 (a)	0.806–0.094	0.826–0.090	a-b	0.799–0.096	0.799–0.096	a-b	0.875–0.139	0.771–0.102	a-b
	2 (b)	0.688–0.118	0.500–0.219		0.417–0.309	0.667–0.248		0.583–0.248	0.417–0.230	
5	1 (a)	0.792–0.097	0.819–0.092	a-b	0.778–0.098	0.799–0.096	a-b	0.726–0.107	0.792–0.097	a-b
	2 (b)	0.580–0.106	0.417–0.230		0.500–0.354	0.417–0.230		0.417–0.230	0.625–0.240	

*Abbreviations*: *SE* standart error, *1st* first, *2nd* second readings; voxel sizes: (1) 0.075 mm^3^, (2) 0.100 mm^3^, (3) 0.150 mm^3^, (4) 0.200 mm^3^, (5) 0.400 mm^3^; implant types: (1) titanium implant, (2) zirconium implant

Same letters indicate statistical significance **≤** 0.005

Figure 4 shows the ROC curves drawn for observers 1, 2, and 3, respectively, for 1st and 2nd readings. The AUC value reflects the area under the ROC curve, with a higher value indicating a higher accuracy. The highest AUC values were obtained with the 0.150 mm^3^.

**Figure:**
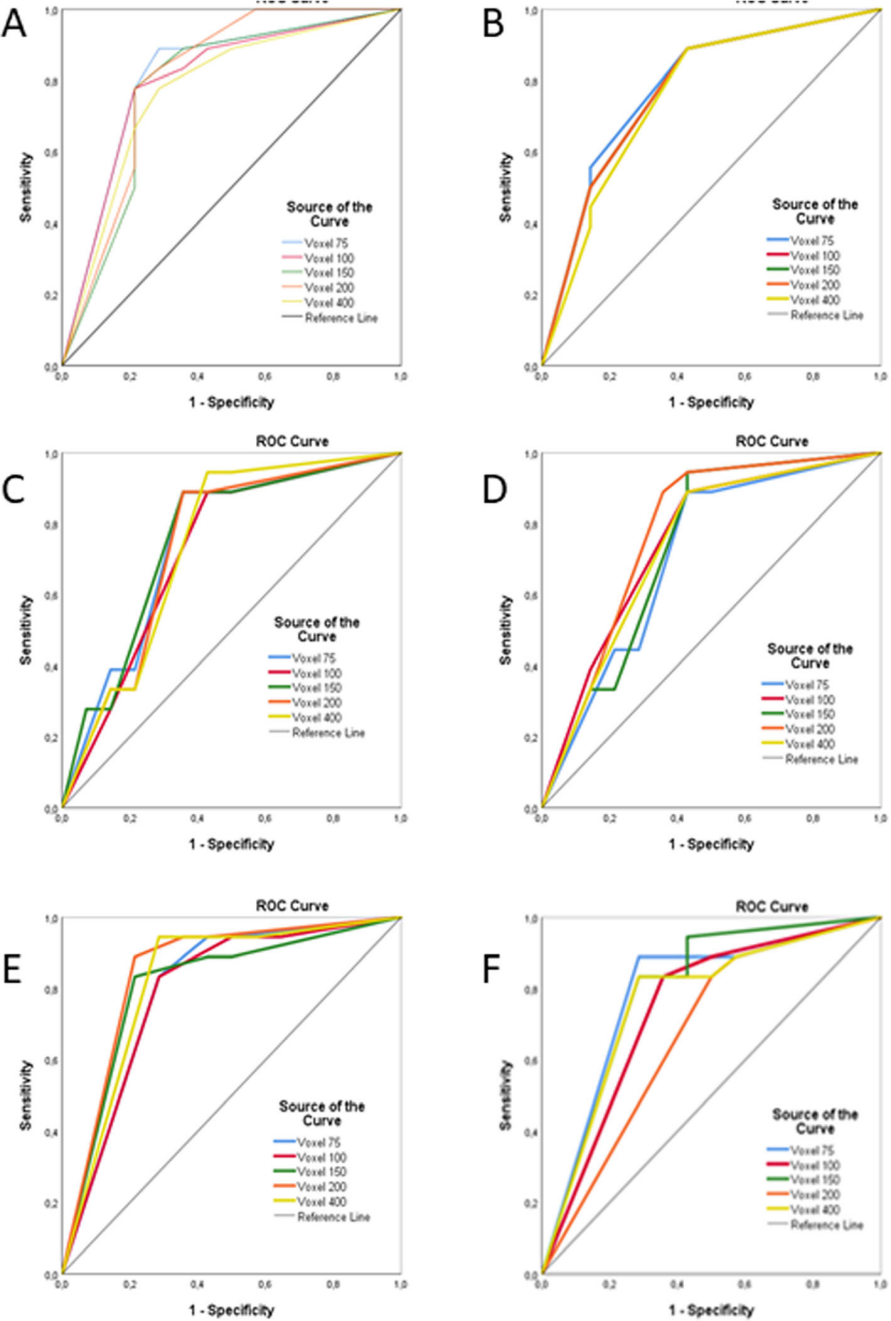
Fig. 4

Table 4 shows the sensitivity, specificity, positive predictive value (PPV), and negative predictive value (NPV) for all observers according to voxel sizes and implants. All voxel sizes had almost similar sensitivity and specificity values while in 0.075–0.400 mm^3^ voxel sizes positive predictive values were lower than 0.200, 0.150 mm^3^. Titanium implants were found higher sensitivity, specificity, PPV, and NPV values for all voxels than zirconium implants.

**Table 4:** Sensitivity, specificity, positive predictive value (PPV), and negative predictive value (NPV) for the observers

		Sensitivity			Specifity			PPV			NPV		
Voxel size	Implant type	Observer 1	Observer 2	Observer 3	Observer 1	Observer 2	Observer 3	Observer 1	Observer 2	Observer 3	Observer 1	Observer 2	Observer 3
		Readings 1st 2nd	Readings 1st 2nd	Readings 1st 2nd	Readings 1st 2nd	Readings 1st 2nd	Readings 1st 2nd	Readings 1st 2nd	Readings 1st 2nd	Readings 1st 2nd	Readings 1st 2nd	Readings 1st 2nd	Readings 1st 2nd
**1**	**1**	0.75–0.66	0.75–0.66	0.66–0.58	0.91–0.91	0.91–0.91	1.00–0.91	0.73–0.76	0.78–0.69	0.74–0.80	0.78–0.73	0.78–0.73	0.75–0.68
	**2**	0.50–0.00	0.50–0.00	0.00–0.50	0.83–0.83	0.83–0.83	0.83–0.83	0.50–0.00	0.00–0.00	0.00–0.50	0.75–0.62	0.71–0.71	0.71–0.83
**2**	**1**	0.75–0.66	0.75–0.66	0.58–0.66	0.83–0.91	0.91–0.91	1.00–0.83	0.78–0.75	0.80–0.79	0.75–0.73	0.76–0.73	0.83–0.71	0.71–0.83
	**2**	0.00–0.00	0.50–0.00	0.00–0.50	0.68–0.73	0.83–0.83	0.83–0.83	0.00–0.00	0.50–0.00	0.00–0.50	0.71–0.71	0.68–0.73	0.69–0.65
**3**	**1**	0.75–0.66	0.66–0.66	0.66–0.58	0.83–0.91	0.91–0.91	0.91–0.83	0.90–0.84	0.93–0.86	0.89–0.76	0.76–0.73	0.73–0.73	0.83–0.83
	**2**	0.50–0.00	0.50–0.00	0.50–0.50	0.68–0.73	0.83–0.83	0.83–0.83	0.50–0.00	0.50–0.00	0.50–0.50	0.83–0.71	0.83–0.71	0.69–0.65
**4**	**1**	0.75–0.66	0.66–0.66	0.66–0.58	0.83–0.91	0.91–0.91	1.00–0.83	0.84–0.77	0.80–0.77	0.76–0.82	0.76–0.73	0.73–0.73	0.83–0.71
	**2**	0.50–0.00	0.50–0.50	0.50–0.00	0.68–0.73	0.83–0.83	0.83–0.83	0.50–0.00	0.50–0.50	0.50–0.00	0.83–0.71	0.83–0.83	0.75–0.66
**5**	**1**	0.75–0.66	0.75–0.66	0.66–0.50	0.83–0.91	0.91–0.91	1.00–0.83	0.67–0.72	0.71–0.70	0.61–0.59	0.76–0.73	0.68–0.73	0.75–0.62
	**2**	0.50–0.00	0.50–0.00	0.00–0.50	0.66–0.83	0.83–0.91	0.83–0.83	0.33–0.00	0.33–0.00	0.00–0.50	0.80–0.71	0.85–0.71	0.71–0.83

*Abbreviations*: *PPV* positive predictive value, *NPV* negative predictive value, *1st* first, *2nd* second; voxel sizes: (1) 0.075 mm^3^, (2) 0.100 mm^3^, (3) 0.150 mm^3^, (4) 0.200 mm^3^, (5) 0.400 mm^3^; implant types: (1) titanium implant, (2) zirconium implant

## Discussion

The absence of cortical plates around the cervical root or implant surfaces leads to alveolar defects such as fenestration. These defects reduce bone support for dental implants and teeth [33, 34]. The reason for the occurrence of defects may be related to the incorrect placement of the implant during surgery, excessive loading, and the inflammation caused by biofilm. Complications related to esthetic and hygiene occur due to the prevention of the defects of the implant surface from being completely overlapped [35].

Radiographs are essential methods for the detection of anatomical structures such as the alveolar bone. Buccal and lingual bone defects cannot examine with 2D imaging modalities. 3D imaging can be considered as a diagnostic tool [36]. Intraoral radiographs have shown 63–67% sensitivity in detection and identification of artificially created bone defects, CBCT has shown 80–100% sensitivity in previous studies [18, 36, 37].

Previous studies have been carried out using CBCT to evaluate dehiscence and fenestrations because it is reliable for evaluation of bone morphology with a 3D imaging [33, 38, 39].

Since voxel size affects diagnostic capacity in CBCT images, there are different results on these reports that investigate the effect of voxel size for detecting disease in the literature. Thus, in the current study, CBCT images with different voxel sizes were used to detect the fenestrations around dental implants.

Ganguly et al. used 0.16 mm^3^, 0.2 mm^3^, and 0.3 mm^3^ voxel size for maxillary and mandibular linear measurements in human cadaver heads then compared physical measurements and find that there was no difference between the linear measurement [40]. Librizzi et al. tested 0.2 mm^3^, 0.3 mm^3^, and 0.4 mm^3^ voxel sizes for detecting erosions of the mandibular condyle and found that 0.2 mm^3^ voxel size more useful [41]. Baltacioğlu et al. determined that there was no difference between 0.150 mm^3^, 0.200 mm^3^, and 0.400 mm^3^ voxel size for detecting recurrent caries [42], contrary to that Haiter et al. concluded that 0.125 mm^3^ voxel size provides more accurate results than 0.160 mm^3^, 0.250 mm^3^, and 0.36 mm^3^ voxel size for approximal caries lesion [43].

In this study depending on the software’s capabilities, all available voxel sizes were tested. It was already indicated that the smaller voxel sizes the larger radiation dose [44]. Therefore, a threshold for the voxel size to detect the fenestrations is thought to be deemed necessary. It was found in the present study, intra-observer agreements were the highest for 0.150 mm^3^ in both titanium and zirconium implants. However, when the voxel sizes decreases down to 0.075 mm^3^, the agreement drops in parallel to voxel size. This issue can be inter-pratede with the occurrence of artifacts around dental implants, as scattering or complete absorption of the beam can exist and be concluded with image degradation. This situation can prevent observation of the implant-bone inter-face and make it difficult to evaluate peri-implant bone defects [45, 46].

Kolsuz et al. asses to influence of voxel size with 0.080 mm^3^, 0.100 mm^3^, 0.125 mm^3^, 0.150 mm^3^, 0.160 mm^3^, and 0.200 mm^3^ for detection periodontal defects and indicated that there is no significant difference between voxel size up to 0.150 mm [36]. Similarly, Bagis et al. used 0.80 mm^3^, 0.125 mm^3^, and 0.160 mm^3^ voxel size and reported that for 0.80 mm^3^ and 0.125 mm^3^ voxel sizes has a greater degree of agreement than 0.160 mm [47].

In our study, we found that the highest kappa value at 0.150 mm^3^ and 0.200 mm^3^ voxel size lower kappa value at 0.400 mm^3^ for all observers and both readings when compare gold standard. These findings are similar to Kolsuz and Bagis’ results [34, 47].

A similar study compared two voxel sizes (0.12 mm and 0.2 mm) and scan modes (180° half scan and 360° full scan) using i-CAT NG unit in the estimation of titanium peri-implant fenestration and dehiscence defects. The authors found that 0.2 mm voxel size had slightly higher diagnostic values than the 0.12 mm voxel size, but there was not a significant difference in detecting bone defects. They concluded that both voxel sizes were similar to detect peri-implant fenestrations and defects [12].. Demirtürk et al. found a significant difference in their study between images acquired with higher resolution (0.2 mm and 0.25 mm voxel sizes) compared with those acquired with lower resolution (0.3 mm and 0.4 mm voxel sizes) with voxel sizes of 0.3 and 0.4 mm producing fewer artifacts. This result is in line with this current study which concluded as the moderate voxel sizes (0.150 or 0.200 mm^3^) showed higher agreement [48]. The present study showed a significant difference in detecting peri-implant fenestrations in different voxel resolutions both in zirconium and titanium implants. The highest agreement observed for 0.150 mm^3^ voxel resolution for both implant types. The smaller voxel sizes the higher radiation dose in line with higher artifact generation. The highest DAP for values achieved in smaller voxel sizes in same FOV. This may due to exposure time (s) and mAs in small voxel sizes. Moreover, images with higher voxel sizes have low-spatial resolutions, and this situation may affect the performance of diagnostic capabilities of the images [49]. The higher diagnostic capability of 0.150 mm^3^ voxel size compared to other voxel sizes can be explained by all this information.

The X-ray attenuation is different from a structure to another due to their atomic number and the presence of high atomic number materials. The higher the atomic number they have, the more artifact expression may be seen increasing the variability of the grey values, leading to a change of the image contrast and decrease the visualization of structures [50]. In order to test differences between dental implants, in this study, two different types of dental implant materials (Ti^22^ and Zr^40^) were used.

Demirtürk et al. also evaluated artifacts generated by zirconium, titanium, and titanium-zirconium alloy implants using different imaging modalities including CBCT in different voxel resolutions. They concluded that they found less artifacts for titanium and titanium-zirconium implants than zirconium implants [48]. Similarly, Bayrak et al. evaluated the same types of implants to detect of peri-implant dehiscences on CBCT images. They found a higher agreement on detection of peri-implant dehiscences for titanium implants than zirconium and both zirconium and titanium implants [51]. These results are in line with the current study which showed lower agreement for zirconium implant, which may interpreted as the nature of its material influence of the diagnosis of fenestration.

The limitations of this study were; first, we did not use different FOV sizes in the CBCT unit.

It is known that FOV size influences CBCT image quality because of the effect of scattered radiation [52]. In a recent study, Vasconcelos et al. assessed the performance of two metal artifact reduction (MAR) algorithms in cone beam computed tomography (CBCT) imaging, considering different materials, metal positions, and fields of view (FOVs). They stated that for ProMax3D, higher standard deviation of voxel gray values in the images acquired with small FOVs in comparison with medium FOVs [53]. Various FOV sizes can affect the quality of the images due to the scatter radiation. In this study, it was attempted to change the voxel sizes concerning FOV to assess the observer agreements as a combination of these.

The second limitation was the lack of optimization filters such as metal artifact reduction (MAR) or adaptive image noise optimizer (AINO) did not test in this study which can dramatically affect the images and reduce the artifacts and enhance image quality.

Bayrak et al. investigated the effect of a metal artifact reduction (MAR) algorithm and the adaptive image noise optimizer (AINO) optimization filter in the evaluation of dehiscences around implants with CBCT in a similar set-up. They concluded that both filters enhanced the ability of detection for the artificially created peri-implant dehiscences. They recommended that the combination of using both filters for detecting peri-implant dehiscences [51].

Vasconcelos et al. also performed a study to assess the effects of different scanning procedures both with and without MAR mode in a ProMax 3D CBCT unit (Planmeca Oy, Helsinki, Finland) to the artifact around zirconium implant. They found that selection MAR mode reduced to artifact value [54]. In this study, the MAR algorithm in this study also reduced the artifact but no statistically significant difference found for both observers in all scan modes. However, it should state that higher agreement Kappa values achieved with the gold standard in titanium implants for all each observer and scan modes.

Since FOV sizes and optimization filters such as MAR or AINO may also affect the diagnosis of peri-implant bone defects together with voxel sizes. Further studies should be carried out to examine the variables.

## Conclusions

A voxel size of 0.150 mm^3^ was identified as the cut-off point for the overall detection of peri-implant fenestrations defects. CBCT should be considered the most reliable imaging modality for the diagnosis of periodontal defects for both zirconium and titanium implants.

## Data Availability

The data used and/or analyzed during the current study are available from the corresponding author on reasonable request.

## Abbreviations

CBCT: Cone beam computed tomography
Zr: Zirconium
Ti: Titanium
AUC: Area under the curve
3D: Three dimensional
2D: Two dimensional
FOV: Field of view
TFT: Thin film transistor
ROC: Receiver operating characteristic
TPR: True-positive rate
FPR: False-positive rate
PPV: Positive predictive value
NPV: Negative predictive value
MAR: Metal artifact reduction
AINO: Adaptive image noise optimizer
DAP: Dose area product
